# Analysis of Outage Probability and Average Bit Error Rate of Parallel-UAV-Based Free-Space Optical Communications

**DOI:** 10.3390/e27060650

**Published:** 2025-06-18

**Authors:** Sheng-Hong Lin, Jin-Yuan Wang, Xinyi Hua

**Affiliations:** 1 Jiangsu Province Service Customization Network Application Engineering Research Center, Nanjing Vocational College of Information Technology, Nanjing 210023, China; 2 School of Communications and Information Engineering, Nanjing University of Posts and Telecommunications, Nanjing 210003, China; jywang@njupt.edu.cn; 3 Elevator Department One, Branch of Wuxi, Jiangsu Province Special Equipment Safety Supervision Inspection Institute, Wuxi 214073, China; huaxy@wxtjy.com

**Keywords:** average bit error rate, FSO communications, outage probability, parallel UAV relays

## Abstract

Recently, free-space optical (FSO) communication systems utilizing unmanned aerial vehicle (UAV) relays have garnered significant attention. Integrating UAV relays into FSO communication and employing cooperative diversity techniques not only fulfill the need for long-distance transmission but also enable flexible adjustments of relay positions based on the actual environment. This paper investigates the performance of a parallel-UAV-relay-based FSO communication system. In the considered system, the channel fadings include atmospheric loss, atmospheric turbulence, pointing errors, and angle-of-arrival fluctuation. Using the established channel model, we derive a tractable expression for the probability density function of the total channel gain. Then, we derive closed-form expressions of the system outage probability (OP) and average bit error rate (ABER). Moreover, we also derive the asymptotic OP and ABER for a high-optical-intensity regime. Our numerical results validate the accuracy of the derived theoretical expressions. Additionally, the effects of the number of relay nodes, the field of view, the direction deviation, the signal-to-noise ratio threshold, the atmospheric turbulence intensity, the transmit power, and the transmission distance on the system’s performance are also discussed.

## 1. Introduction

### 1.1. Background and Motivation

Recently, free-space optical (FSO) communication has drawn widespread attention because of its inherent advantages, which include strong confidentiality, no requirement for spectrum licensing, and a high communication capacity [1,2]. However, FSO signals are highly susceptible to various fadings, such as atmospheric turbulence, atmospheric loss, and pointing errors [3]. Moreover, the performance of FSO systems is severely limited by the strict line-of-sight (LoS) alignment requirements between their transmitter and receiver [4]. To address these limitations, relay-assisted FSO communication has emerged as a promising solution [5], which is where relays are strategically deployed between the transmitter and the receiver to improve the overall performance of the system. Currently, most terrestrial FSO systems utilize fixed relays. However, due to potential obstructions in the environment in which they are deployed, the optimal placement of these relays is often unattainable, thereby restricting their practical applicability.

As unmanned aerial vehicle (UAV) technology develops rapidly, UAV relay-based FSO communication systems have received increasing amounts of attention [6]. Unlike traditional fixed terrestrial relays, UAV-assisted relays can easily establish LoS links by dynamically adjusting their positions, which can effectively address the challenges associated with relay deployment. Recently, UAV-relay-based FSO communication has become a promising candidate in many research fields, such as emergency communication and military operations [7,8,9]. In this paper, we will analyze the key performance indicators of a parallel-UAV-relay-based FSO communication system. This analysis will enable the rapid performance evaluation of such systems without the need for time-consuming simulations.

### 1.2. Related Works

In terms of FSO communication systems based on UAV relays, current related research primarily focuses on channel modeling and system parameter optimization. A tractable performance indicator expression is crucial for an effective system performance analysis. Specifically, the outage probability (OP) of an FSO system under a log-normal channel was analyzed [10]. An integral form of the OP for the multi-hop FSO communication system was obtained [11]. In [12], the authors analyzed the throughput of a UAV-based hybrid FSO/radio frequency (RF) system with buffer constraints. In [13], the authors studied the performance of a dual-hop fixed-gain FSO relaying system with differential detection and direct detection. In [14], a theoretical expression of the OP for FSO communication systems based on a Markov chain was obtained. However, these studies [10,11,12,13,14] fail to account for the impact of the angle of arrival (AoA) fluctuations resulting from the orientation deviations of hovering UAVs. In [15], a multi-rotor UAV-based air channel was established by comprehensively considering path loss, atmospheric turbulence, pointing error, and AoA fluctuation. For FSO communication with a high-altitude UAV, the weak atmospheric turbulence, path loss, pointing deviation, and AoA fluctuation were considered while modeling the UAV-to-UAV link [16]. In [17], an FSO channel model with weak turbulence, pointing errors, and AoA fluctuation was considered. For the multi-rotor UAV relay-based air channel, the AoA fluctuation was also discussed to demonstrate the impact of the UAV jitter [18]. However, the derived system performance expressions in [15,16,17,18] are cumbersome and intractable. Therefore, to obtain further insights, it is necessary to develop tractable theoretical expressions.

Moreover, the majority of current research on UAV-based FSO communication has primarily focused on serial relaying. For instance, in a serial UAV-based multi-hop relaying FSO system, exact and asymptotic expressions of OP were derived [19]. The performance of a triple-hop FSO system with amplification-and-forward relaying has been analyzed [20]. In [21], a regenerate-and-forward relaying scheme was proposed for an FSO serial relay-assisted system to expand its transmit distance. Using the decode-and-forward (DF) scheme, the authors of another paper optimized key parameters to minimize the outage probability of a UAV-based FSO system [22]. The throughput of a dual-hop FSO/RF hybrid system based on serial UAV relays, was maximized in [23]. When multiple UAV relays exist in the system and cooperate and communicate, the use of parallel relays can further enhance the overall performance of the system. However, the performance of a parallel-UAV-relay-based FSO system has not been extensively investigated in the available literature.

### 1.3. Contributions and Organization

Motivated by the issues mentioned above, this paper focuses on the error performance analysis of an FSO communication system based on parallel UAV relays. The main focus in this paper is error probability, which is a key aspect of reliable communication in Claude Shannon’s information theory. The main contributions of this paper are as follows:

- We establish a system model for parallel-UAV-relay-based FSO communication systems. We consider an FSO communication scenario with a source node, *M* relay nodes, and a destination node. The received signal and signal-to-noise ratio (SNR) are analyzed. We establish an FSO channel model that considers atmospheric loss, atmospheric turbulence, pointing error, and AoA fluctuation.
- We analyze the performance of a system for parallel-UAV-relay-based FSO communication. For the considered system, we obtain tractable closed-form expressions of the OP and average bit error rate (ABER). Our numerical results indicate that the simulation results are in good agreement with the theoretical results, which verifies the correctness of the expressions we derived.
- We analyze the asymptotic performance of the considered system. The theoretical expressions of the asymptotic OP and asymptotic ABER are derived at high optical powers. Through analysis, it is found that the asymptotic OP is restricted by the number of relay nodes, the field of view (FoV) of the receiver, and the standard deviation of direction deviation. However, the asymptotic ABER is only related to the FoV and the standard deviation of the direction deviation, and is independent of the number of relay nodes.

The rest of this paper is organized as follows: Section 2 provides the system model. In Section 3, closed-form expressions of the OP and ABER are derived, and the asymptotic performance of the system is also analyzed. Section 4 presents several numerical results. Finally, the conclusions are provided in Section 5.

*Notation*: Throughout this paper, regular font indicates a scalar. Γ(·) is the Gamma function; $K_{n}\left(\cdot \right)$ is the modified Bessel function of the second kind with order *n* [24]; δ(·) is the Dirac delta function; $G_{p,q}^{m,n}\left[\cdot \right]$ is Meijer’s G-function [24]; Pr(·) is the probability of an event; $F_{\gamma_{i}}\left(\cdot \right)$ is the cumulative distribution function (CDF) of $\gamma_{i}$; and $\mathrm{erfc}\left(x\right)=2\int_{x}^{\infty }e^{-t^{2}}dt/\sqrt{\pi }$ is the complementary error function.

## 2. System and Channel Models

### 2.1. System Model

**Figure 1:**
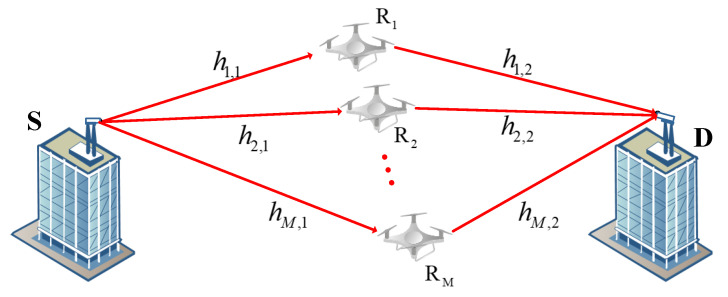
Model of FSO communication system based on parallel UAV relays.

We consider a parallel-UAV-relay-based FSO system consisting of a source node (S), *M* relay nodes ($R_{1},R_{2},\ldots ,R_{M}$), and a destination node (D), as illustrated in Figure 1. In this system, node S and node D are installed on the top of buildings, and hovering UAVs are used as the relay nodes. We suppose that the direct transmission link between node S and node D is blocked. Moreover, node S uses laser diodes as its transmitter to send optical signals; each UAV relay node employs the DF protocol and node D uses photodiodes to receive the signal and perform photoelectric conversion.

The entire communication system includes *M* parallel paths, and each path contains two hops. For the first hop, node S transmits the same signal $x_{s}$ to *M* UAV relay nodes; the signal received by UAV relay node $R_{m}$ can be written as

(1) $$y_{m,1}=Th_{m,1}x_{s}+n_{m},\;\forall m\in \left\{1,2,\ldots ,M\right\}$$

where *T* denotes the photoelectric conversion efficiency of the receiver’s photodiode at $R_{m}$, $h_{m,1}$ is the channel gain between node S and node $R_{m}$, and $n_{m}$ is the additive white Gaussian noise (AWGN) at $R_{m}$ with mean zero and variance $\sigma_{r}^{2}$.

In addition, the transmitted signal consists of symbols that are randomly selected with equal probability from the on–off keying (OOK) constellation such that $x_{s}\in \left\{0,2P_{t}\right\}$, where $P_{t}$ denotes the average optical power of transmission. After that, the received electrical SNR of the *m*th link is given by

(2) $$\gamma_{m,1}=\frac{2P_{t}^{2}T^{2}}{\sigma_{r}^{2}}h_{m,1}^{2}$$

For the second hop, the node $R_{m}$ adopts the DF protocol. That is, the received signal is first decoded, and then the re-encoded signal $x_{r}$ is sent to node D. Therefore, the signal received at node D is expressed as

(3) $$y_{m,2}=Th_{m,2}x_{r}+n_{d},\;\forall m\in \left\{1,2,\ldots ,M\right\}$$

where $h_{m,2}$ is the channel gain between node $R_{m}$ and node D and $n_{d}$ is the AWGN at node D with mean zero and variance $\sigma_{d}^{2}$.

Similarly, the transmitted signal at each UAV relay is also considered as symbols were selected with equal probability from the OOK constellation such that $x_{r}\in \left\{0,2P_{o}\right\}$, where $P_{o}$ is the average transmit power of each relay. Then, the SNR $\gamma_{m,2}$ of the second hop is given by

(4) $$\gamma_{m,2}=\frac{2P_{o}^{2}T^{2}}{\sigma_{d}^{2}}h_{m,2}^{2}$$

For each path, the end-to-end SNR $\gamma_{m}$ can be written as

(5) $$\gamma_{m}=\min\left\{\gamma_{m,1},\gamma_{m,2}\right\}$$

To combine the received signal, a selection combining (SC) receiver is used. Compared to other combining schemes, the SC receiver is easier to implement since only one path is used for transmission. Moreover, the SC receiver is more robust in dealing with channel estimation errors since the weakest paths are excluded from the combining process. Therefore, the total SNR γ of the system can be expressed as

(6) $$\gamma =\max\left\{\gamma_{1},\gamma_{2},\ldots ,\gamma_{M}\right\}$$

### 2.2. Channel Model

In this paper, the channel gain $h_{m,j},\;m\in \left\{1,\ldots ,M\right\},j\in \left\{1,2\right\}$ is mainly influenced by four factors [19], i.e.,

(7) $$h_{m,j}=h_{m,j}^{l}h_{m,j}^{a}h_{m,j}^{p}h_{m,j}^{\mathrm{aoa}}$$

where $h_{m,j}^{1}$ is atmospheric loss, $h_{m,j}^{a}$ is atmospheric turbulence, $h_{m,j}^{p}$ is pointing error, and $h_{m,j}^{\mathrm{aoa}}$ is AoA fluctuation.

According to the exponential Beers–Lambert law, the atmospheric loss can be modeled as [19]

(8) $$h_{m,j}^{1}=\exp\left(-Z_{m,j}\xi \right)$$

where $Z_{m,j}$ is the length of the *j*th hop of the *m*th path and ξ represents the atmospheric attenuation coefficient.

For atmospheric turbulence, the Gamma–Gamma distribution is employed since it is suitable for all turbulence conditions [25]. The probability density function (PDF) of $h_{m,j}^{a}$ can be expressed as [26]

(9) $$f_{h_{m,j}^{a}}\left(h_{m,j}^{a}\right)=\frac{2{\left(\alpha_{m,j}\beta_{m,j}\right)}^{\frac{\alpha_{m,j}+\beta_{m,j}}{2}}}{\Gamma \left(\alpha_{m,j}\right)\Gamma \left(\beta_{m,j}\right)}{\left(h_{m,j}^{a}\right)}^{\frac{\alpha_{m,j}+\beta_{m,j}}{2}-1}K_{\alpha_{m,j}-\beta_{m,j}}\left(2\sqrt{\alpha_{m,j}\beta_{m,j}h_{m,j}^{a}}\right),h_{m,j}^{a}>0$$

where $\alpha_{m,j}$ and $\beta_{m,j}$ are the effective numbers of large-scale and small-scale eddies in the scattering process and given by

(10) αm,j=exp0.49σR,m,j20.49σR,m,j21+1.11σR,m,j12/51+1.11σR,m,j12/57/6−1−1βm,j=exp0.51σR,m,j20.51σR,m,j21+0.69σR,m,j12/51+0.69σR,m,j12/55/6−1−1

where $\sigma_{R,m,j}^{2}=1.23C_{n}^{2}k^{7/6}Z_{m,j}^{11/6}$ denotes the Rytov variance, $C_{n}^{2}$ denotes the index of refraction structure parameter, k=2π2πλλ denotes the number of light waves, and λ denotes the wavelength.

Based on [10], we assume that the random variances in position at each UAV are Gaussian-distributed with zero mean and variance $\sigma_{p,u}^{2}$, the random variances in the direction deviation of the UAVs are Gaussian-distributed with zero mean and variance $\sigma_{o}^{2}$, and the random variances in position deviation at the ground platform are Gaussian-distributed with zero mean and variance $\sigma_{p,g}^{2}$. Therefore, the PDF of the pointing error is written as

(11) $$f_{h_{m,j}^{p}}\left(h_{m,j}^{p}\right)=\frac{\zeta_{m,j}^{2}}{A_{m,j}^{\zeta_{m,j}^{2}}}{\left(h_{m,j}^{p}\right)}^{\zeta_{m,j}^{2}-1},0\leq h_{m,j}^{p}\leq A_{m,j}$$

where ζm,j2=ωZeq,m,j2ωZeq,m,j24σs,m,j24σs,m,j2; $A_{m,j}={\left[\mathrm{erf}\left(v_{m,j}\right)\right]}^{2}$ is the maximal fraction of collected power, with vm,j=πam,jπam,j2ωZm,j2ωZm,j; $a_{m,j}$ is the aperture radius; $\omega_{Z_{m,j}}$ is the beam waist radius at distance $Z_{m,j}$; and ωZeq,m,j2=ωZm,j2πerfvm,jωZm,j2πerfvm,j2vm,jexp−vm,j22vm,jexp−vm,j2 is the equivalent beam waist. Moreover, $\sigma_{s,m,j}^{2}$ is the variance in the total displacement for different links, which is given by

(12) $$\sigma_{s,m,j}^{2}=\left\{\begin{array}{c} \sigma_{p,g}^{2}+\sigma_{p,u}^{2},j=1\\ \sigma_{p,g}^{2}+\sigma_{p,u}^{2}+Z_{m,j}^{2}\sigma_{o}^{2},j=2 \end{array}\right.$$

From [9,27], it is known that the link between transmitter and receiver is interrupted when the AoA of the received beam falls outside the FoV of the receiver. The PDF of $h_{m,j}^{\mathrm{aoa}}$ can be modeled as

(13) $$f_{h_{m,j}^{\mathrm{aoa}}}\left(h_{m,j}^{\mathrm{aoa}}\right)=\exp\left(-\frac{\theta_{\mathrm{FoV},m,j}^{2}}{2\sigma_{o}^{2}}\right)\delta \left(h_{m,j}^{\mathrm{aoa}}\right)+\left[1-\exp\left(-\frac{\theta_{\mathrm{FoV},m,j}^{2}}{2\sigma_{o}^{2}}\right)\right]\delta \left(h_{m,j}^{\mathrm{aoa}}-1\right)$$

where $\theta_{\mathrm{FoV},m,j}$ is the FoV of the *m*th receiver at the *j*th hop and $\sigma_{o}^{2}$ is the variance in the direction deviation at each UAV.

According to (8), (9), (11), and (13), the PDF of $h_{m,j}$ is derived as follows:

(14) $$\begin{array}{cc} f_{h_{m,j}}\left(h_{m,j}\right) & =\exp\left(-\frac{\theta_{\mathrm{FoV},m,j}^{2}}{2\sigma_{o}^{2}}\right)\delta \left(h_{m,j}\right)+\left[1-\exp\left(-\frac{\theta_{\mathrm{FoV},m,j}^{2}}{2\sigma_{o}^{2}}\right)\right]\\ \; & \times \frac{\alpha_{m,j}\beta_{m,j}\zeta_{m,j}^{2}}{A_{m,j}h_{m,j}^{l}\Gamma \left(\alpha_{m,j}\right)\Gamma \left(\beta_{m,j}\right)}\\ \; & \times G_{1,3}^{3,0}\left[\frac{\alpha_{m,j}\beta_{m,j}}{A_{m,j}h_{m,j}^{l}}h_{m,j}\left|\begin{array}{c} \zeta_{m,j}^{2}\\ \zeta_{m,j}^{2}-1,\alpha_{m,j}-1,\beta_{m,j}-1 \end{array}\right.\right],h_{m,j}\geq 0 \end{array}$$

## 3. Performance Analysis

This section will analyze the OP and ABER of the parallel-UAV-relay-based FSO communication system before carrying out an asymptotic analysis of the system’s performance.

### 3.1. Outage Probability

The OP is a common metric for measuring the reliability of a wireless communication system. The OP is defined as the probability of the received SNR γ being smaller than a given threshold $\gamma_{\mathrm{th}}$. Therefore, the OP can be expressed as

(15) $$\begin{array}{cc} P_{\mathrm{out}}\left(\gamma_{\mathrm{th}}\right)\; & =\mathrm{Pr}\left(\gamma \leq \gamma_{\mathrm{th}}\right)\\ \; & =\mathrm{Pr}\left(\max\left\{\gamma_{1},\gamma_{2},\ldots ,\gamma_{M}\right\}\leq \gamma_{\mathrm{th}}\right)\\ \; & =\prod_{m=1}^{M}F_{\gamma_{m}}\left(\gamma_{\mathrm{th}}\right) \end{array}$$

where $F_{\gamma_{m}}\left(\cdot \right)$ represents the CDF of $\gamma_{m}$. Note that the last equality holds because the *M* paths are independent of each other.

According to (5), $F_{\gamma_{m}}\left(\gamma_{\mathrm{th}}\right)$ can be expressed as

(16) $$F_{\gamma_{m}}\left(\gamma_{\mathrm{th}}\right)=1-\left[1-F_{\gamma_{m,1}}\left(\gamma_{\mathrm{th}}\right)\right]\left[1-F_{\gamma_{m,2}}\left(\gamma_{\mathrm{th}}\right)\right]$$

where $F_{\gamma_{m,1}}\left(\cdot \right)$ and $F_{\gamma_{m,2}}\left(\cdot \right)$ represent the CDFs of $\gamma_{m,1}$ and $\gamma_{m,2}$, respectively.

According to (2), (14), and (9.31.5) in [24], $F_{\gamma_{m,1}}\left(\gamma_{\mathrm{th}}\right)$ can be further written as

(17) $$\begin{array}{cc} F_{\gamma_{m,1}}\left(\gamma_{\mathrm{th}}\right) & =F_{h_{m,1}}\left(h_{\mathrm{th}}\right)\\ \; & =\exp\left(-\frac{\theta_{\mathrm{FoV},m,1}^{2}}{2\sigma_{o}^{2}}\right)+\left[1-\exp\left(-\frac{\theta_{\mathrm{FoV},m,1}^{2}}{2\sigma_{o}^{2}}\right)\right]\frac{\zeta_{m,1}^{2}}{\Gamma \left(\alpha_{m,1}\right)\Gamma \left(\beta_{m,1}\right)}\\ \; & \times G_{2,4}^{3,1}\left[\frac{\alpha_{m,1}\beta_{m,1}}{A_{m,1}h_{m,1}^{l}}\sqrt{\frac{\gamma_{\mathrm{th}}\sigma_{r}^{2}}{2T^{2}P_{t}^{2}}}\left|\begin{array}{c} 1,\zeta_{m,1}^{2}+1\\ \zeta_{m,1}^{2},\alpha_{m,1},\beta_{m,1},0 \end{array}\right.\right] \end{array}$$

where hth=γthσr2γthσr22T2Pt22T2Pt2 is the corresponding threshold of the channel gain.

Similarly, the expression of $F_{\gamma_{m,2}}\left(\gamma_{\mathrm{th}}\right)$ can be obtained as

(18) $$\begin{array}{cc} F_{\gamma_{m,2}}\left(\gamma_{\mathrm{th}}\right) & =\exp\left(-\frac{\theta_{\mathrm{FoV},m,2}^{2}}{2\sigma_{o}^{2}}\right)+\left[1-\exp\left(-\frac{\theta_{\mathrm{FoV},m,2}^{2}}{2\sigma_{o}^{2}}\right)\right]\frac{\zeta_{m,2}^{2}}{\Gamma \left(\alpha_{m,2}\right)\Gamma \left(\beta_{m,2}\right)}\\ \; & \times G_{2,4}^{3,1}\left[\frac{\alpha_{m,2}\beta_{m,2}}{A_{m,2}h_{m,2}^{l}}\sqrt{\frac{\gamma_{\mathrm{th}}\sigma_{d}^{2}}{2T^{2}P_{o}^{2}}}\left|\begin{array}{c} 1,\zeta_{m,2}^{2}+1\\ \zeta_{m,2}^{2},\alpha_{m,2},\beta_{m,2},0 \end{array}\right.\right] \end{array}$$

Substituting (17) and (18) into (16), we can obtain the following expression of $F_{\gamma_{m}}\left(\gamma_{\mathrm{th}}\right)$:

(19) $$\begin{array}{cc} F_{\gamma_{m}}\left(\gamma_{\mathrm{th}}\right) & =1-\left\{\left[1-\exp\left(-\frac{\theta_{\mathrm{FoV},m,1}^{2}}{2\sigma_{o}^{2}}\right)\right]\left[1-\frac{\zeta_{m,1}^{2}}{\Gamma \left(\alpha_{m,1}\right)\Gamma \left(\beta_{m,1}\right)}\right.\right.\\ \; & \left.\left.\times G_{2,4}^{3,1}\left[\frac{\alpha_{m,1}\beta_{m,1}}{A_{m,1}h_{m,1}^{l}}\sqrt{\frac{\gamma_{\mathrm{th}}\sigma_{r}^{2}}{2T^{2}P_{t}^{2}}}\left|\begin{array}{c} 1,\zeta_{m,1}^{2}+1\\ \zeta_{m,1}^{2},\alpha_{m,1},\beta_{m,1},0 \end{array}\right.\right]\right]\right\}\\ \; & \times \left\{\left[1-\exp\left(-\frac{\theta_{\mathrm{FoV},m,2}^{2}}{2\sigma_{o}^{2}}\right)\right]\left[1-\frac{\zeta_{m,2}^{2}}{\Gamma \left(\alpha_{m,2}\right)\Gamma \left(\beta_{m,2}\right)}\right.\right.\\ \; & \left.\left.\times G_{2,4}^{3,1}\left[\frac{\alpha_{m,2}\beta_{m,2}}{A_{m,2}h_{m,2}^{l}}\sqrt{\frac{\gamma_{\mathrm{th}}\sigma_{d}^{2}}{2T^{2}P_{o}^{2}}}\left|\begin{array}{c} 1,\zeta_{m,2}^{2}+1\\ \zeta_{m,2}^{2},\alpha_{m,2},\beta_{m,2},0 \end{array}\right.\right]\right]\right\} \end{array}$$

Finally, by substituting (19) into (15), we can derive a closed-form expression for the OP of the system as

(20) $$\begin{array}{cc} P_{\mathrm{out}}\left(\gamma_{\mathrm{th}}\right) & =\prod_{m=1}^{M}\left\{1-\left\{\left[1-\exp\left(-\frac{\theta_{\mathrm{FoV},m,1}^{2}}{2\sigma_{o}^{2}}\right)\right]\left\{1-\frac{\zeta_{m,1}^{2}}{\Gamma \left(\alpha_{m,1}\right)\Gamma \left(\beta_{m,1}\right)}\right.\right.\right.\\ \; & \left.\left.\times G_{2,4}^{3,1}\left[\frac{\alpha_{m,1}\beta_{m,1}}{A_{m,1}h_{m,1}^{l}}\sqrt{\frac{\gamma_{\mathrm{th}}\sigma_{r}^{2}}{2T^{2}P_{t}^{2}}}\left|\begin{array}{c} 1,\zeta_{m,1}^{2}+1\\ \zeta_{m,1}^{2},\alpha_{m,1},\beta_{m,1},0 \end{array}\right.\right]\right\}\right\}\\ \; & \times \left\{\left[1-\exp\left(-\frac{\theta_{\mathrm{FoV},m,2}^{2}}{2\sigma_{o}^{2}}\right)\right]\left\{1-\frac{\zeta_{m,2}^{2}}{\Gamma \left(\alpha_{m,2}\right)\Gamma \left(\beta_{m,2}\right)}\right.\right.\\ \; & \left.\left.\left.\times G_{2,4}^{3,1}\left[\frac{\alpha_{m,2}\beta_{m,2}}{A_{m,2}h_{m,2}^{l}}\sqrt{\frac{\gamma_{\mathrm{th}}\sigma_{d}^{2}}{2T^{2}P_{o}^{2}}}\left|\begin{array}{c} 1,\zeta_{m,2}^{2}+1\\ \zeta_{m,2}^{2},\alpha_{m,2},\beta_{m,2},0 \end{array}\right.\right]\right\}\right\}\right\} \end{array}$$

### 3.2. Asymptotic Outage Probability

In this subsection, the asymptotic OP will be analyzed when the average transmitted optical power $P_{t}$ at node S and the average optical power $P_{o}$ at each relay are sufficiently large.

When $P_{t}$ tends to infinity, the corresponding channel gain threshold $h_{\mathrm{th}}$ will approach zero. According to the series expression of Meijer’s G-function [28], we can simplify (17) to

(21) $$\begin{array}{cc} F_{\gamma_{m,1}}\left(\gamma_{\mathrm{th}}\right) & \approx \exp\left(-\frac{\theta_{\mathrm{FoV},m,1}^{2}}{2\sigma_{o}^{2}}\right)+\left[1-\exp\left(-\frac{\theta_{\mathrm{FoV},m,1}^{2}}{2\sigma_{o}^{2}}\right)\right]\\ \; & \times \frac{\zeta_{m,1}^{2}\Gamma \left(\alpha_{m,1}-\eta_{m,1}\right)b_{m,1}}{\Gamma \left(\alpha_{m,1}\right)\Gamma \left(\beta_{m,1}\right)\eta_{m,1}}{\left(\frac{\alpha_{m,1}\beta_{m,1}}{A_{m,1}h_{m,1}^{l}}\sqrt{\frac{\gamma_{\mathrm{th}}\sigma_{r}^{2}}{2T^{2}P_{t}^{2}}}\right)}^{\eta_{m,1}},\eta_{m,1}=\min\left(\zeta_{m,1}^{2},\beta_{m,1}\right) \end{array}$$

where bm,1=11ζm,12−βm,1ζm,12−βm,1, if $\zeta_{m,1}^{2}>\beta_{m,1}$; and $b_{m,1}=\Gamma \left(\beta_{m,1}-\zeta_{m,1}^{2}\right)$, if $\zeta_{m,1}^{2}<\beta_{m,1}$.

In (21), the term containing γthσr2γthσr22T2Pt22T2Pt2 becomes zero when $P_{t}$ tends to infinity. Thus, the asymptotic expression of $F_{\gamma_{m,1}}\left(\gamma_{\mathrm{th}}\right)$ can be expressed as

(22) $$\begin{array}{c} F_{\gamma_{m,1}}^{\mathrm{bound}}\left(\gamma_{\mathrm{th}}\right)=\exp\left(-\frac{\theta_{\mathrm{FoV},m,1}^{2}}{2\sigma_{o}^{2}}\right) \end{array}$$

Similarly, when $P_{o}\to \infty$ in (18), we can derive the asymptotic expression of $F_{\gamma_{m,2}}\left(\gamma_{\mathrm{th}}\right)$ as

(23) $$\begin{array}{c} F_{\gamma_{m,2}}^{\mathrm{bound}}\left(\gamma_{\mathrm{th}}\right)=\exp\left(-\frac{\theta_{\mathrm{FoV},m,2}^{2}}{2\sigma_{o}^{2}}\right) \end{array}$$

Substituting (22) and (23) into (16), we can derive the asymptotic bound of $F_{\gamma_{m}}\left(\gamma_{\mathrm{th}}\right)$ as

(24) $$\begin{array}{cc} F_{\gamma_{m}}^{\mathrm{bound}}\left(\gamma_{\mathrm{th}}\right) & =1-\left[1-\exp\left(-\frac{\theta_{\mathrm{FoV},m,1}^{2}}{2\sigma_{o}^{2}}\right)\right]\left[1-\exp\left(-\frac{\theta_{\mathrm{FoV},m,2}^{2}}{2\sigma_{o}^{2}}\right)\right]\\ \; & =1-\prod_{j=1}^{2}\left[1-\exp\left(-\frac{\theta_{\mathrm{FoV},m,j}^{2}}{2\sigma_{o}^{2}}\right)\right] \end{array}$$

Finally, substituting (24) into (15), we can derive the asymptotic expression of the system OP as

(25) $$\begin{array}{c} P_{\mathrm{out}}^{\mathrm{bound}}\left(\gamma_{\mathrm{th}}\right)=\prod_{m=1}^{M}\left\{1-\prod_{j=1}^{2}\left[1-\exp\left(-\frac{\theta_{\mathrm{FoV},m,j}^{2}}{2\sigma_{o}^{2}}\right)\right]\right\} \end{array}$$

**Remark** **1.** *By observing (25), it can be found that when the average transmit optical power $P_{t}$ and $P_{o}$ are sufficiently large, increasing the optical power does not further improve the outage performance of the system. In this case, the OP of the system is only related to the number of UAV relay nodes M, FoV $\theta_{\mathrm{FoV},m,j}$, and the standard deviation of the direction deviation $\sigma_{o}$.*

### 3.3. Average Bit Error Rate

The ABER is another metric commonly used to measure the performance of communication systems. It reflects the average accuracy of the data transmitted by the considered wireless communication system under all possible channel states.

For the considered system, the end-to-end ABER is related to the ABER of each path, which is their minimum bit error value. In particular, when all paths are independent and identically distributed, the end-to-end ABER of the system is equal to the ABER of a single path. In this case, the ABER of the system can be derived.

Assuming that the receiver uses OOK modulation and direct detection, the end-to-end BER conditioned on the channel gain $h_{m,j}$ for the mth path is expressed as

(26) $$P_{e\left|h_{m,j}\right.}\left(j\right)=\mathrm{Pr}\left(1\right)\mathrm{Pr}\left(e\left|1\right.,h_{m,j}\right)+\mathrm{Pr}\left(0\right)\mathrm{Pr}\left(e\left|0\right.,h_{m,j}\right),j=1,2$$

where $\mathrm{Pr}\left(0\right)$ and $\mathrm{Pr}\left(1\right)$ are the probabilities that the transmitted power is 0 and $2P_{j}$, respectively.

Since $\mathrm{Pr}\left(0\right)=\mathrm{Pr}\left(1\right)=1/2$ and $\mathrm{Pr}\left(e\left|0\right.,h_{m,j}\right)=\mathrm{Pr}\left(e\left|1\right.,h_{m,j}\right)$, Equation (26) can be further written as

(27) $$P_{e\left|h_{m,j}\right.}\left(j\right)=\frac{1}{2}\mathrm{erfc}\left(\frac{P_{j}Th_{m,j}}{\sqrt{2}\sigma_{j}}\right),j=1,2$$

where $P_{j}$ is the average transmit optical power of node S or each relay and $\sigma_{j}$ is the standard variance of noise at each relay or node D. Specifically, when j=1, $P_{j}=P_{t}$ and $\sigma_{j}=\sigma_{r}$; when j=2, $P_{j}=P_{o}$ and $\sigma_{j}=\sigma_{d}$.

According to the following equality [29]

(28) $$\mathrm{erfc}\left(x\right)=\frac{1}{\sqrt{\pi }}G_{1,2}^{2,0}\left[x^{2}\left|\begin{array}{c} 1\\ 0,\frac{1}{2} \end{array}\right.\right]$$

Equation (27) can be rewritten as

(29) $$P_{e\left|h_{m,j}\right.}\left(j\right)=\mathrm{erfc}\left(\frac{P_{j}Th_{m,j}}{\sqrt{2}\sigma_{j}}\right)\frac{1}{2\sqrt{\pi }}G_{1,2}^{2,0}\left[\frac{P_{j}^{2}T^{2}h_{m,j}^{2}}{2\sigma_{j}^{2}}\left|\begin{array}{c} 1\\ 0,\frac{1}{2} \end{array}\right.\right],j=1,2$$

Due to the effect of channel fading, the *j*th hop’s ABER can be expressed as

(30) $$P_{e}\left(j\right)=\int_{0}^{\infty }f_{h_{m,j}}\left(h_{m,j}\right)P_{e\left|h_{m,j}\right.}\left(j\right)dh_{m,j}$$

where $P_{e}\left(1\right)$ denotes the ABER of the first hop (i.e., the source-to-relay link) and $P_{e}\left(2\right)$ denotes the ABER of the second hop (i.e., the relay-to-destination link).

Substituting (14) and (29) into (30), we can obtain the expression for the *j*th hop ABER, which is as follows:

(31) $$\begin{array}{ccc} & & P_{e}\left(j\right)=\frac{1}{2}\exp\left(-\frac{\theta_{\mathrm{FoV},m,j}^{2}}{2\sigma_{o}^{2}}\right)+\frac{2^{\alpha_{m,j}+\beta_{m,j}-4}\zeta_{m,j}^{2}}{\sqrt{\pi^{3}}\Gamma \left(\alpha_{m,j}\right)\Gamma \left(\beta_{m,j}\right)}\left[1-\exp\left(-\frac{\theta_{\mathrm{FoV},m,j}^{2}}{2\sigma_{o}^{2}}\right)\right]\\ & & \times G_{6,3}^{2,5}\left[\frac{8{\left(A_{m,j}h_{m,j}^{l}P_{j}T\right)}^{2}}{\alpha_{m,j}^{2}\beta_{m,j}^{2}\sigma_{j}^{2}}\left|\begin{array}{c} \frac{2-\zeta_{m,j}^{2}}{2},\frac{1-\alpha_{m,j}}{2},\frac{2-\alpha_{m,j}}{2},\frac{1-\beta_{m,j}}{2},\frac{2-\beta_{m,j}}{2},1\\ 0,\frac{1}{2},\frac{-\zeta_{m,j}^{2}}{2} \end{array}\right.\right],j=1,2 \end{array}$$

According to [30], the ABER of the dual-hop system meets the following conditions:

(32) $$\left\{\begin{array}{c} P_{e,j}=\left(1-P_{e,j-1}\right)P_{e}\left(j\right)+P_{e,j-1}\left(1-P_{e}\left(j\right)\right),j=1,2\\ P_{e,0}=0 \end{array}\right.$$

where $P_{e,j}$ is the ABER from the source node to the relay node (j=1) or destination node (j=2).

Thus, the total ABER from node S to node D can be expressed as

(33) $$P_{e,2}=P_{e}\left(1\right)+P_{e}\left(2\right)-2P_{e}\left(1\right)P_{e}\left(2\right)$$

Substituting (31) into (33), the total ABER $P_{e}$ (i.e., $P_{e,2}$) can be obtained as

(34) $$\begin{array}{cc} P_{e} & =\sum_{j=1}^{2}\left\{\frac{1}{2}\exp\left(-\frac{\theta_{\mathrm{FoV},m,j}^{2}}{2\sigma_{o}^{2}}\right)+\frac{2^{\alpha_{m,j}+\beta_{m,j}-4}\zeta_{m,j}^{2}}{\sqrt{\pi^{3}}\Gamma \left(\alpha_{m,j}\right)\Gamma \left(\beta_{m,j}\right)}\left[1-\exp\left(-\frac{\theta_{\mathrm{FoV},m,j}^{2}}{2\sigma_{o}^{2}}\right)\right]\right.\\ \; & \left.\times G_{6,3}^{2,5}\left[\frac{8{\left(A_{m,j}h_{m,j}^{l}P_{j}T\right)}^{2}}{\alpha_{m,j}^{2}\beta_{m,j}^{2}\sigma_{j}^{2}}\left|\begin{array}{c} \frac{2-\zeta_{m,j}^{2}}{2},\frac{1-\alpha_{m,j}}{2},\frac{2-\alpha_{m,j}}{2},\frac{1-\beta_{m,j}}{2},\frac{2-\beta_{m,j}}{2},1\\ 0,\frac{1}{2},\frac{-\zeta_{m,j}^{2}}{2} \end{array}\right.\right]\right\}\\ \; & -2\prod_{j=1}^{2}\left\{\frac{1}{2}\exp\left(-\frac{\theta_{\mathrm{FoV},m,j}^{2}}{2\sigma_{o}^{2}}\right)+\frac{2^{\alpha_{m,j}+\beta_{m,j}-4}\zeta_{m,j}^{2}}{\sqrt{\pi^{3}}\Gamma \left(\alpha_{m,j}\right)\Gamma \left(\beta_{m,j}\right)}\left[1-\exp\left(-\frac{\theta_{\mathrm{FoV},m,j}^{2}}{2\sigma_{o}^{2}}\right)\right]\right.\\ \; & \left.\times G_{6,3}^{2,5}\left[\frac{8{\left(A_{m,j}h_{m,j}^{l}P_{j}T\right)}^{2}}{\alpha_{m,j}^{2}\beta_{m,j}^{2}\sigma_{j}^{2}}\left|\begin{array}{c} \frac{2-\zeta_{m,j}^{2}}{2},\frac{1-\alpha_{m,j}}{2},\frac{2-\alpha_{m,j}}{2},\frac{1-\beta_{m,j}}{2},\frac{2-\beta_{m,j}}{2},1\\ 0,\frac{1}{2},\frac{-\zeta_{m,j}^{2}}{2} \end{array}\right.\right]\right\} \end{array}$$

### 3.4. Asymptotic Average BER

This subsection will derive the asymptotic bound of the ABER when the average transmit optical power $P_{j}$ is sufficiently large.

According to the series expression of Meijer’s G-function [24], when $P_{j}$ tends to infinity, we can express (31) as

(35) $$\begin{array}{cc} P_{e}\left(j\right) & \approx \frac{1}{2}\exp\left(-\frac{\theta_{\mathrm{FoV},m,j}^{2}}{2\sigma_{o}^{2}}\right)+\frac{2^{\alpha_{m,i}+\beta_{m,j}-4}\zeta_{m,j}^{2}}{\sqrt{\pi^{3}}\Gamma \left(\alpha_{m,j}\right)\Gamma \left(\beta_{m,j}\right)}\left[1-\exp\left(-\frac{\theta_{\mathrm{FoV},m,j}^{2}}{2\sigma_{o}^{2}}\right)\right]\\ \;\;\; & \times \frac{2\Gamma \left(\frac{1+\alpha_{m,j}-\psi_{m,j}}{2}\right)\Gamma \left(\frac{\alpha_{m,j}-\psi_{m,j}}{2}\right)\Gamma \left(\frac{1+\psi_{m,j}}{2}\right)d_{m,j}}{\psi_{m,j}}\\ \;\;\; & \times {\left(\frac{\alpha_{m,j}^{2}\beta_{m,j}^{2}\sigma_{j}^{2}}{8{\left(A_{m,j}h_{m,j}^{l}P_{j}T\right)}^{2}}\right)}^{\frac{\psi_{m,j}}{2}},\psi_{m,j}=\min\left(\zeta_{m,j}^{2},\beta_{m,j}\right) \end{array}$$

where dm,j=Γβm,j−ζm,j2βm,j−ζm,j222Γ1+βm,j−ζm,j21+βm,j−ζm,j222, if $\zeta_{m,j}^{2}<\beta_{m,j}$. Moreover, dm,j=2π2πζm,j2−βm,jζm,j2−βm,j if $\zeta_{m,j}^{2}>\beta_{m,j}$.

In (35), when the average transmitted optical power $P_{j}$ tends to infinity, the term containing $P_{j}$ tends to zero. Thus, the asymptotic expression of $P_{e}\left(j\right)$ can be expressed as

(36) $$P^{\mathrm{bound}}\left(j\right)=\frac{1}{2}\exp\left(-\frac{\theta_{\mathrm{FoV},m,j}^{2}}{2\sigma_{o}^{2}}\right)$$

Substituting (36) into (34), we can obtain the asymptotic expression of the ABER as

(37) $$P_{e}^{\mathrm{bound}}=\frac{1}{2}\sum_{j=1}^{2}\exp\left(-\frac{\theta_{\mathrm{FoV},m,j}^{2}}{2\sigma_{o}^{2}}\right)-\frac{1}{2}\prod_{j=1}^{2}\exp\left(-\frac{\theta_{\mathrm{FoV},m,j}^{2}}{2\sigma_{o}^{2}}\right)$$

**Remark** **2.** *In (37), when $P_{j}\to \infty$, increasing $P_{j}$ will not further improve the ABER of the system. In this situation, the ABER of the system is only related to the FoV $\theta_{\mathrm{FoV},m,j}$ and the standard deviation of direction deviation $\sigma_{o}$ and is independent of the number of relay nodes.*

## 4. Numerical Results

In this section, Monte-Carlo simulations will be provided to verify the correctness of the theoretical expressions we have derived, and the impacts of various parameters on the system’s performance will be provided. In all simulation figures, the theoretical results of the OP and asymptotic OP are determined by (20) and (25), respectively. The theoretical results of the ABER and asymptotic ABER are determined by (34) and (37), respectively. To evaluate Meijer’s G-function in our theoretical expressions, we use the software MATLAB R2023a. To facilitate the simulation, the optical power transmitted at node S and each relay are assumed to be the same, i.e., $P_{t}=P_{o}$. The main simulation parameters used in this paper are shown in Table 1.

### 4.1. OP Results

**Figure 2:**
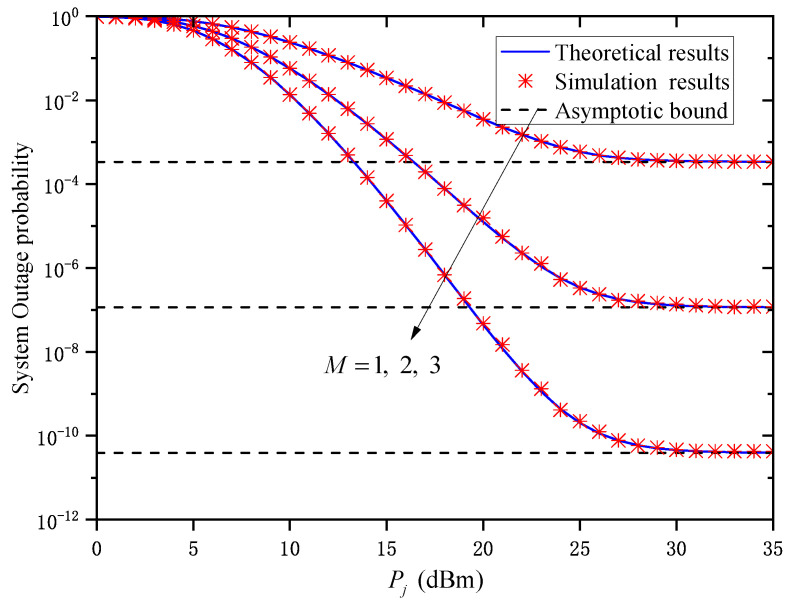
System OP versus $P_{j}$ under different *M* when $Z_{m,j}=0.5\;\mathrm{km}$ and $\sigma_{R,m,j}^{2}=1$.

Figure 2 shows the OP versus the average transmitted optical power $P_{j}$ for different relay numbers *M* when $Z_{m,j}=0.5\;\mathrm{km}$ and $\sigma_{R,m,j}^{2}=1$. As can be observed, for a given *M*, the OP gradually decreases with the $P_{j}$. Under the same $P_{j}$, the OP decreases significantly with an increase in *M*. This means that increasing the number of parallel relay nodes can significantly improve the system’s outage performance. In addition, when $P_{j}$ is large, the OP of the system does not decrease with the increase in $P_{j}$ but tends to the corresponding asymptotic bound. This indicates that increasing the average transmitted optical power cannot always improve the outage performance of the system, which verifies the conclusion in Remark 1.

**Figure 3:**
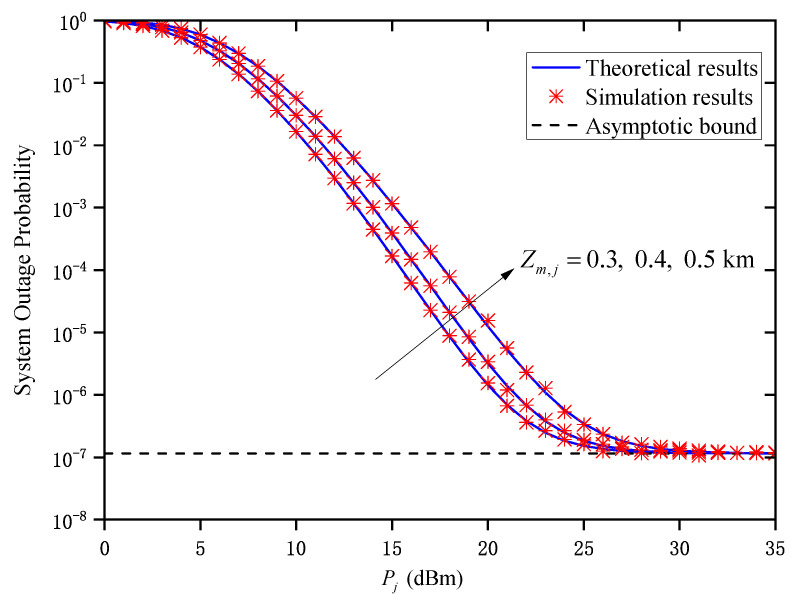
System OP versus $P_{j}$ under different $Z_{m,j}$ when M=2 and $\sigma_{R,m,j}^{2}=1$.

Figure 3 shows the OP versus $P_{j}$ for different $Z_{m,j}$ when M=2 and $\sigma_{R,m,j}^{2}=1$. As can be seen, when $P_{j}$ is small, the system’s OP gradually decreases with the decrease in $Z_{m,j}$. Obviously, the smaller the link transmission distance is, the better the channel quality is. However, when $P_{j}$ is sufficiently large, the OP does not continuously decrease with the increase in $P_{j}$ but gradually approaches its asymptotic bound, which verifies the accuracy of (25). Such an observation suggests that when $P_{j}$ is sufficiently large, the average transmitted optical power will no longer affect the OP performance of the system. In this situation, the relay numbers *M*, the FoV $\theta_{\mathrm{FoV},m,j}$, and the standard deviation of the direction deviation $\sigma_{o}$ limit the system’s outage performance. In addition, when $P_{j}$ is large, the system’s OP curves tend to the same asymptotic bound under different $Z_{m,j}$. That is to say, its asymptotic bound is independent of $Z_{m,j}$. Therefore, the limiting factor affecting the system’s OP is no longer $Z_{m,j}$, which verifies the conclusion in Remark 1.

**Figure 4:**
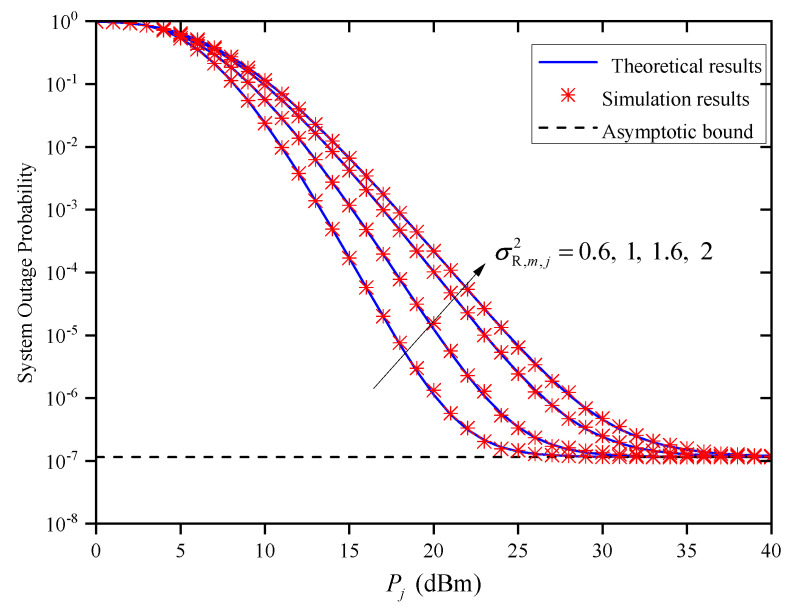
System OP versus $P_{j}$ under different $\sigma_{R,m,j}^{2}$ when M=2 and $Z_{m,j}=0.5\;\mathrm{km}$.

Figure 4 shows the OP versus $P_{j}$ for different $\sigma_{R,m,j}^{2}$ when M=2 and $Z_{m,j}=0.5\;\mathrm{km}$. As can be observed, when $P_{j}$ is small, the OP performance becomes worse as $\sigma_{R,m,j}^{2}$ increases. By observing (9) and (10), we can conclude that the PDF of $h_{m,j}^{a}$ is only affected by $\sigma_{R,m,j}^{2}$. Therefore, when $P_{j}$ is small, the effect of atmospheric turbulence on the OP is reduced by reducing $\sigma_{R,m,j}^{2}$. When $P_{j}$ is large, the OP curves of the system tend to the same asymptotic bound under different $\sigma_{R,m,j}^{2}$. It should be noted that the asymptotic value is independent of $P_{j}$ and the turbulence condition of $\sigma_{R,m,j}^{2}$, which verifies Remark 1.

**Figure 5:**
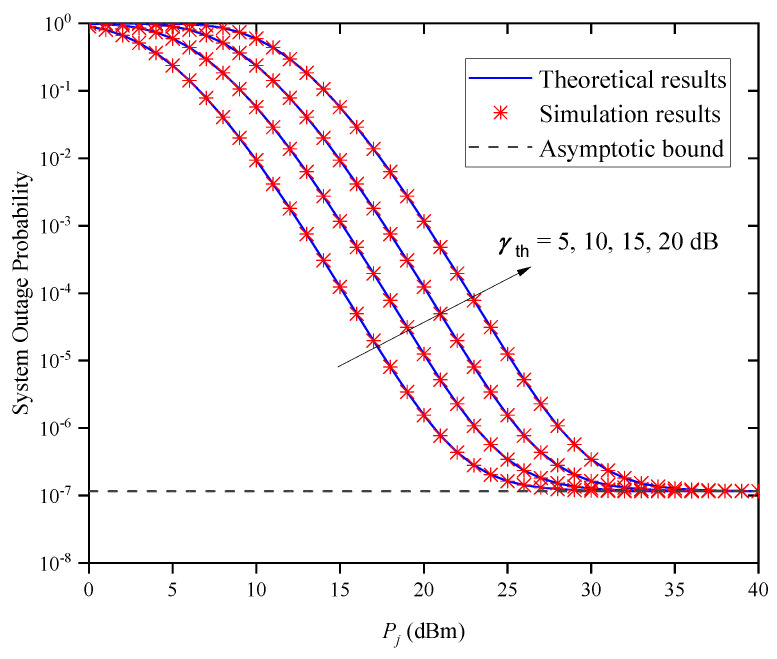
System OP versus average transmitted optical power $P_{j}$ for different $\gamma_{\mathrm{th}}$ when M=2 and $Z_{m,j}=0.5\;\mathrm{km}$.

Figure 5 shows the OP versus $P_{j}$ for different SNR threshold values $\gamma_{\mathrm{th}}$ when M=2 and $Z_{m,j}=0.5\;\mathrm{km}$. It can be observed from the figure that when $P_{j}$ is small, the system’s OP performance becomes worse as the $\gamma_{\mathrm{th}}$ increases, which suggests that a better OP performance for the system can be obtained by reducing the value of $\gamma_{\mathrm{th}}$. In addition, when $P_{j}$ is large, the OP curves of the system tend to the same asymptotic bound, which is independent of $P_{j}$ and the SNR threshold $\gamma_{\mathrm{th}}$, thus verifying Remark 1.

**Figure 6:**
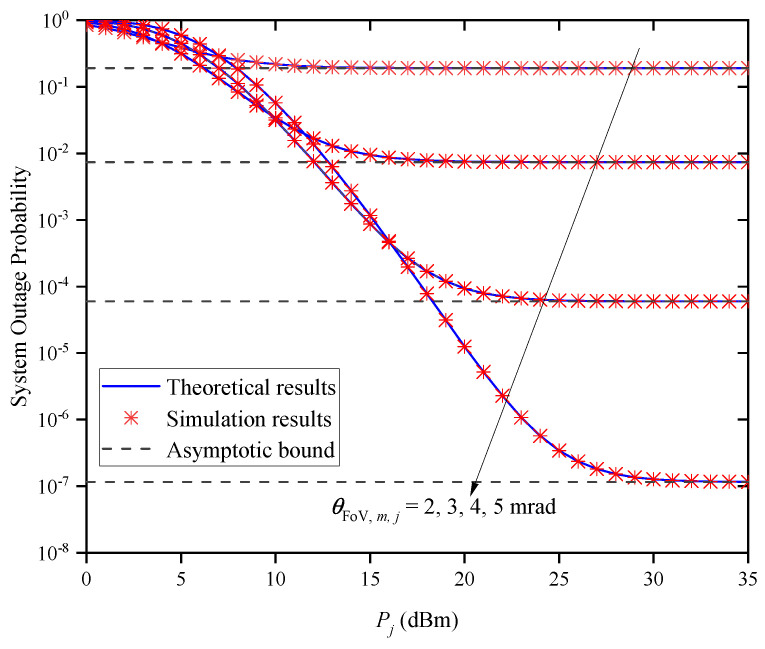
System OP versus $P_{j}$ for different $\theta_{\mathrm{FoV},m,j}$ when M=2 and $Z_{m,j}=0.5\;\mathrm{km}$.

Figure 6 shows the OP versus $P_{j}$ for different $\theta_{\mathrm{FoV},m,j}$ when M=2 and $Z_{m,j}=0.5\;\mathrm{km}$. As seen in the figure, the OP gradually decreases as $P_{j}$ is increased for a given $\theta_{\mathrm{FoV},m,j}$. However, when $P_{j}$ is large, the OP performance does not improve as $P_{j}$ increases, but tends to different asymptotic bounds due to different $\theta_{\mathrm{FoV},m,j}$ values. This indicates that the system’s OP performance cannot always be improved by increasing $P_{j}$ without limitation, which verifies the accuracy of Remark 1.

**Figure 7:**
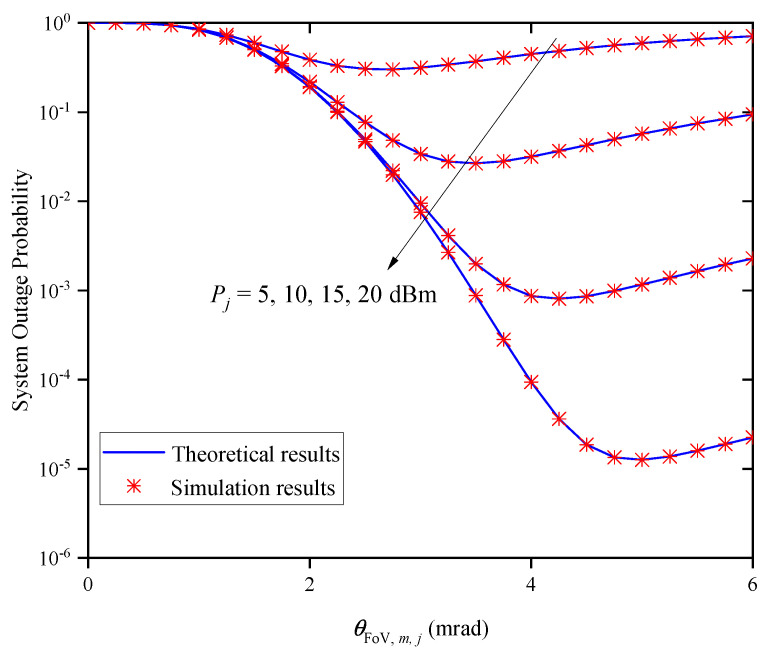
System OP versus FoV for different $P_{j}$ when M=2 and $Z_{m,j}=0.5\;\mathrm{km}$.

Figure 7 shows the OP versus the FoV $\theta_{\mathrm{FoV},m,j}$ for different average transmitted optical powers $P_{j}$ when M=2 and $Z_{m,j}=0.5\;\mathrm{km}$. It can be observed that the OP decreases first and then increases as the $\theta_{\mathrm{FoV},m,j}$ increases for a given $P_{j}$. This indicates that there is an optimal $\theta_{\mathrm{FoV},m,j}$ value for obtaining the best outage performance, and the optimal $\theta_{\mathrm{FoV},m,j}$ value gradually increases with the increase in $P_{j}$.

**Figure 8:**
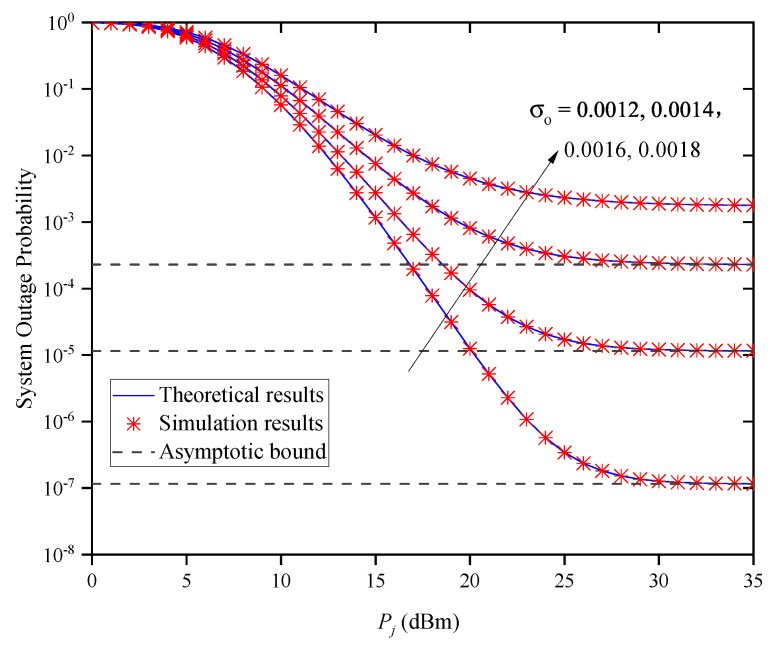
System OP versus $P_{j}$ for different $\sigma_{o}$ when M=2 and $Z_{m,j}=0.5\;\mathrm{km}$.

Figure 8 shows the OP versus $P_{j}$ for different $\sigma_{o}$ when M=2 and $Z_{m,j}=0.5\;\mathrm{km}$. In this figure, the OP gradually decreases with the increase in $P_{j}$ for a fixed $\sigma_{o}$. When the transmitted optical power conditions are the same, the system OP increases with the increase in $\sigma_{o}$, which suggests that a better OP performance for the system can be obtained by decreasing the value of $\sigma_{o}$. Moreover, when $P_{j}$ is large, the OP performance does not decrease with the increase in $P_{j}$ but approaches different asymptotic bounds due to different $\sigma_{o}$ values. This indicates that the system’s OP performance cannot be enhanced by increasing $P_{j}$ without limitation, which verifies the accuracy of Remark 1.

From Figure 2, Figure 3, Figure 4, Figure 5, Figure 6, Figure 7 and Figure 8, it can be observed that the performance gaps between all theoretical and simulation results are so small that they can be ignored. This indicates that the obtained theoretical expression of the OP can be utilized to evaluate a system’s outage performance without the need for time-intensive simulations.

### 4.2. ABER Results

**Figure 9:**
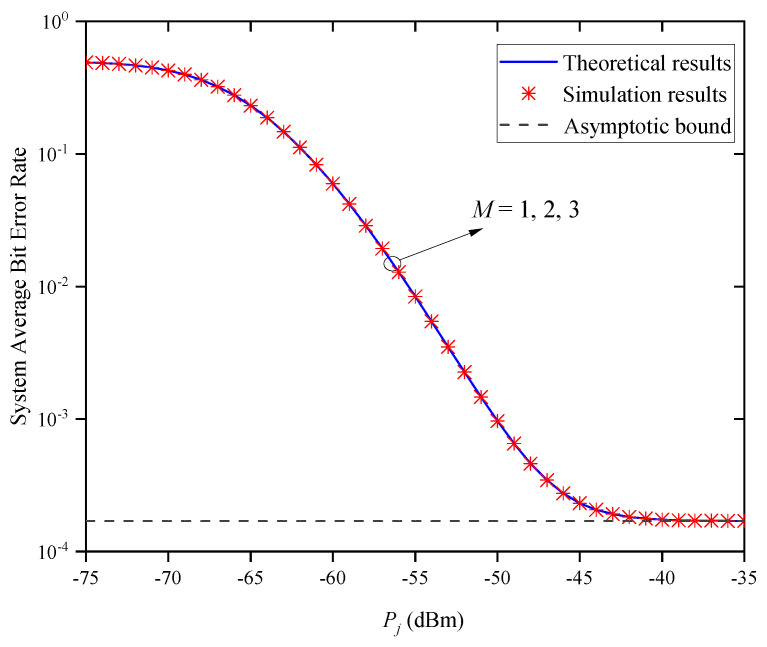
System ABER versus $P_{j}$ for different *M* when $Z_{m,j}=0.5\;\mathrm{km}$ and $\sigma_{R,m,j}^{2}=1$.

Figure 9 shows the system’s ABER versus $P_{j}$ for different numbers of relay nodes *M* when $Z_{m,j}=0.5\;\mathrm{km}$ and $\sigma_{R,m,j}^{2}=1$. It can be seen that the system’s ABER gradually decreases with the increase in $P_{j}$ for a given *M* value. Under the same transmitted optical power condition, the system’s ABER does not change with the increase in *M*, because the system ABER is equal to the ABER of a single path and is independent of *M*, when the states of each path are independent and identically distributed. Moreover, the system ABER does not decrease with the increase in $P_{j}$, but approaches the same asymptotic bound. This indicates that increasing the $P_{j}$ cannot always improve the system’s ABER performance, which verifies the accuracy of Remark 2.

**Figure 10:**
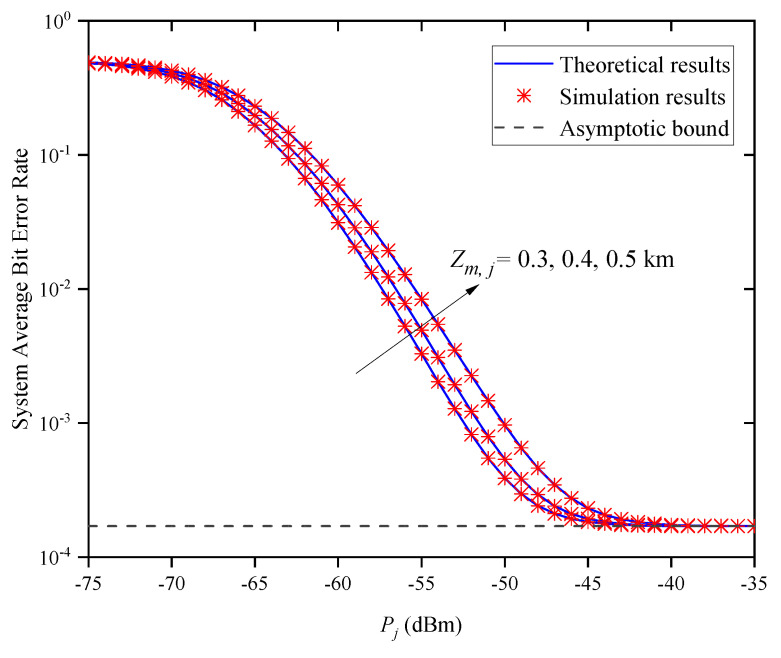
System ABER versus $P_{j}$ for different $Z_{m,j}$ when $\sigma_{R,m,j}^{2}=1$ and M=2.

Figure 10 shows the system’s ABER versus $P_{j}$ for different $Z_{m,j}$ when $\sigma_{R,m,j}^{2}=1$ and M=2. In Figure 10, the system’s ABER dramatically decreases with the decrease in $Z_{m,j}$ when $P_{j}$ is small. This is obvious because the shorter the link transmission distance is, the better the channel quality is. However, when $P_{j}$ is large, the system’s ABER does not continuously decrease with the increase in $P_{j}$, but gradually approaches the asymptotic bound, which verifies the accuracy of (37). This phenomenon suggests that when $P_{j}$ is large enough, the average transmitted optical power does not affect the system’s ABER. Moreover, the system’s ABER curves approach the same asymptotic bound when $P_{j}$ is large; that is, the asymptotic bound is independent of $Z_{m,j}$ and the parameter that affects the system’s ABER is no longer $Z_{m,j}$, which verifies the accuracy of Remark 2.

**Figure 11:**
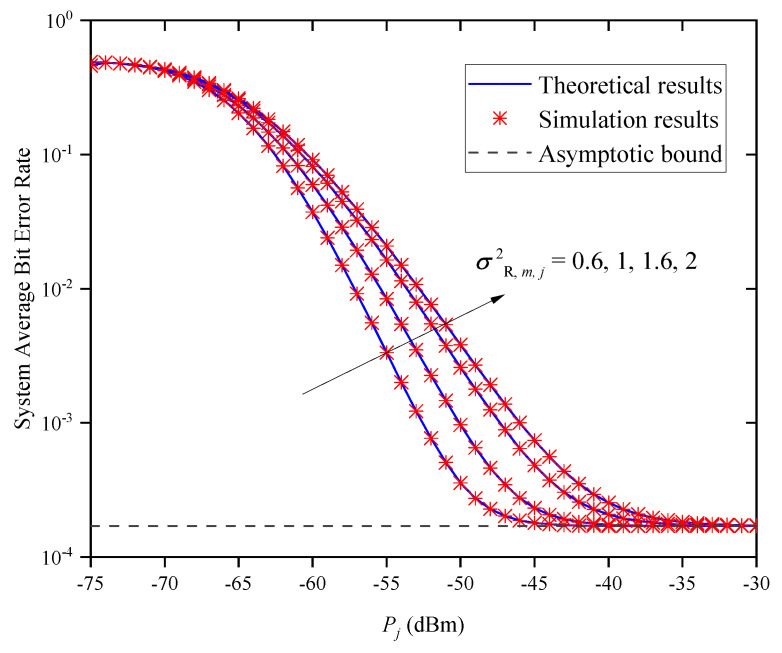
System ABER versus $P_{j}$ for different $\sigma_{R,m,j}^{2}$ when M=2 and $Z_{m,j}=0.5\;\mathrm{km}$.

Figure 11 shows the system’s ABER versus $P_{j}$ for different $\sigma_{R,m,j}^{2}$ when M=2 and $Z_{m,j}=0.5\;\mathrm{km}$. In the figure, we find that the system BER increases with the increase in $\sigma_{R,m,j}^{2}$ when $P_{j}$ is small. According to (9) and (10), we can conclude that the PDF of $h_{m,j}^{a}$ is only determined by $\sigma_{R,m,j}^{2}$. Thus, when the average transmitted optical power is small, we can reduce the effect of atmospheric turbulence on the system’s BER by decreasing $\sigma_{R,m,j}^{2}$. Under different $\sigma_{R,m,j}^{2}$ conditions, the system’s BER curves approach the same asymptotic bound as when the average transmitted optical power is large; this asymptotic value is independent of $P_{j}$ and the turbulence condition $\sigma_{R,m,j}^{2}$, which verifies the accuracy of Remark 2.

**Figure 12:**
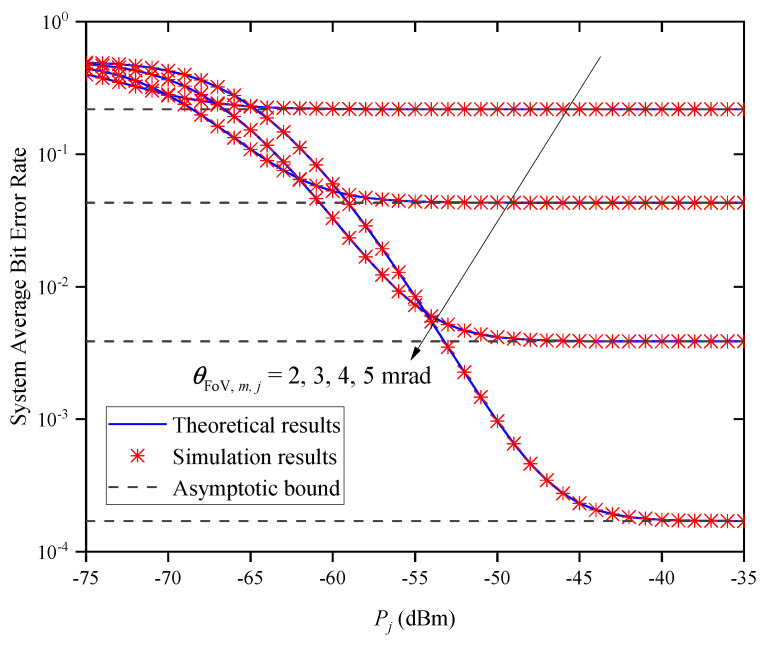
System ABER versus $P_{j}$ for different $\theta_{\mathrm{FoV},m,j}$ when M=2 and $Z_{m,j}=0.5\;\mathrm{km}$.

Figure 12 shows the system’s ABER versus $P_{j}$ for different FoVs $\theta_{\mathrm{FoV},m,j}$ when M=2 and $Z_{m,j}=0.5\;\mathrm{km}$. In the figure, we find that the system’s ABER gradually decreases with the increase in $P_{j}$ for a given $\theta_{\mathrm{FoV},m,j}$. When $P_{j}$ is large, the system’s ABER does not decrease with the increase in $P_{j}$, but approaches different asymptotic bounds due to the different values of $\theta_{\mathrm{FoV},m,j}$. This indicates that the system’s ABER cannot always be decreased by increasing $P_{j}$, which verifies the accuracy of Remark 2.

**Figure 13:**
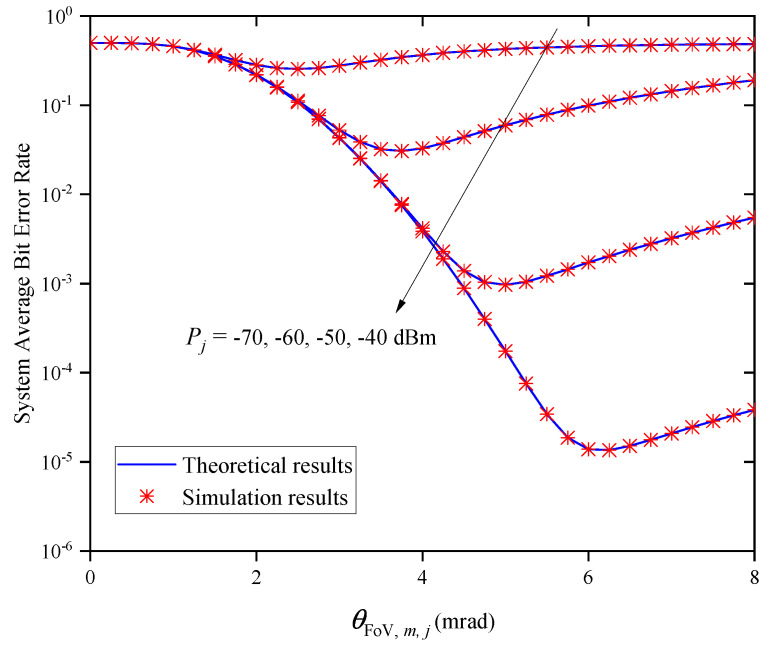
System ABER versus FoV for different $P_{j}$ when M=2 and $Z_{m,j}=0.5\;\mathrm{km}$.

Figure 13 shows the system’s ABER versus the FoV $\theta_{\mathrm{FoV},m,j}$ for different values of $P_{j}$ when M=2 and $Z_{m,j}=0.5\;\mathrm{km}$. In this figure, we find that the system’s ABER decreases first and then increases as $\theta_{\mathrm{FoV},m,j}$ increases for a given $P_{j}$. This indicates that there is an optimal $\theta_{\mathrm{FoV},m,j}$ value for obtaining the minimum system ABER, and the optimal $\theta_{\mathrm{FoV},m,j}$ value increases gradually with the increase in $P_{j}$.

**Figure 14:**
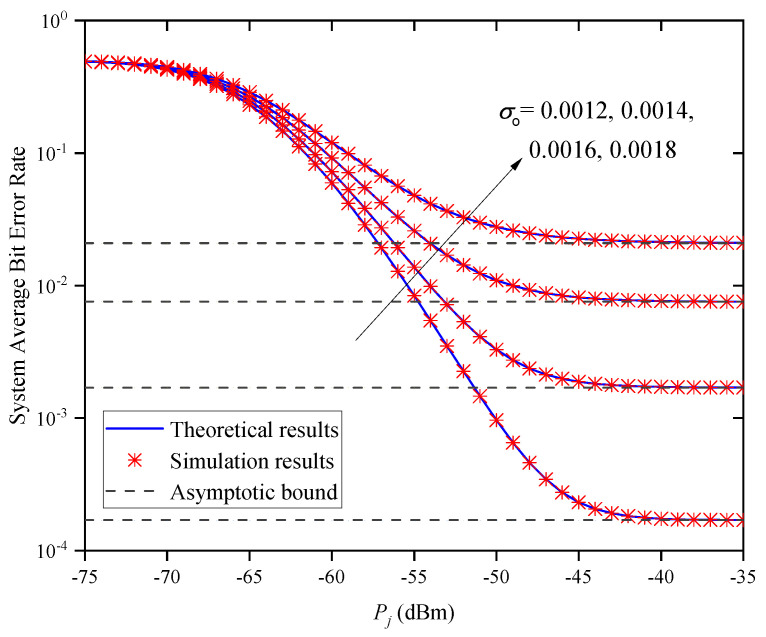
System ABER versus average transmitted optical power for different $\sigma_{o}$ when M=2 and $Z_{m,j}=0.5\;\mathrm{km}$.

Figure 14 shows the system ABER versus $P_{j}$ for different standard deviations of the direction deviation $\sigma_{o}$ when M=2 and $Z_{m,j}=0.5\;\mathrm{km}$. In this figure, we find that the system’s ABER decreases with the increase in $P_{j}$ for a given $\sigma_{o}$. Under the different $P_{j}$ conditions, the system’s ABER increases with the increase in $\sigma_{o}$. This indicates that we can obtain a smaller ABER by reducing the value of $\sigma_{o}$. In addition, when $P_{j}$ is large, the system’s ABER does not decrease with the increase in $P_{j}$ but approaches different asymptotic bounds because of different values of $\sigma_{o}$. This indicates that the system’s ABER cannot always be decreased by increasing $P_{j}$, which verifies the accuracy of Remark 2.

From Figure 9, Figure 10, Figure 11, Figure 12, Figure 13 and Figure 14, we can conclude that all theoretical values match the simulation values well. Thus, the obtained ABER expression could be directly employed to evaluate the system’s error performance without the need for time-consuming simulations.

## 5. Conclusions

In this work, we investigated the outage and error performance of a parallel-UAV-relay-based FSO communication system. By considering the atmospheric loss, atmospheric turbulence, pointing error, and AoA fluctuation, we derived the PDF of the overall channel gain and closed-form expressions of the OP and the ABER. Moreover, we also analyzed the asymptotic bounds of the OP and ABER. Our numerical results verified the accuracy of the theoretical expressions we derived. Moreover, the results indicated that the system’s OP is related to the number of relay nodes, the FoV, and the standard deviation of the direction deviation, while the ABER is only related to the FoV and the standard deviation of the direction deviation.

## Figures and Tables

**Table 1:** The main parameters used in this paper.

Parameters	Symbols	Values
Wavelength	λ	1550 nm
Detector radius	*a*	0.025 m
Refractive index structure coefficient	$C_{n}^{2}$	5×10−14m−2−233
Standard deviation of UAV position deviation	$\sigma_{p,u}$	0.1 m
Photoelectric conversion efficiency	*T*	0.9
Standard deviation of ground position	$\sigma_{p,g}$	0.1 m
Atmospheric attenuation coefficient	ξ	$1\;km^{-1}$
Standard deviation of direction deviation	$\sigma_{o}$	1.2 mrad
Noise variance at $R_{j}$	$\sigma_{r}^{2}$	$2.5\times 10^{-14}$
SNR threshold	$\gamma_{\mathrm{th}}$	10 dB
Noise variance at D	$\sigma_{d}^{2}$	$2.5\times 10^{-14}$
Beam waist radius at distance $Z_{m,j}$	$\omega_{Z_{m,j}}$	2m
Receiver FoV	$\theta_{\mathrm{FoV},m,j}$	5 mrad
Receiver sensitivity	$S_{v}$	−30 dBm

## Data Availability

The data presented in this study are available on request from the corresponding author.

## Abbreviations

The following abbreviations are used in this manuscript:

ABER	average bit error rate
AoA	angle of arrival
AWGN	additive white Gaussian noise
CDF	cumulative distribution function
DF	decode-and-forward
FoV	field of view
FSO	free-space optical
OOK	on–off keying
OP	outage probability
PDF	probability density function
RF	radio frequency
SC	selection combining
SNR	signal-to-noise ratio
UAV	unmanned aerial vehicle
